# Healing patient, harming planet? A drive towards sustainable surgery: review of waste production and recyclability of surgical instrument packaging

**DOI:** 10.1308/rcsann.2023.0045

**Published:** 2024-02-16

**Authors:** YK Lee, A Hariri, R Ghedia, T Tikka, D Kim

**Affiliations:** The Royal Marsden NHS Foundation Trust, UK

**Keywords:** Recycling, Product packaging, Sustainable development, Waste management, Surgery, Thyroidectomy

## Abstract

**Introduction:**

Healthcare contributes more than 1% of all domestic waste in the United Kingdom (UK), with operating theatre waste alone accounting for approximately 50% of all hospital waste. In November 2022, the UK Surgical Royal Colleges issued an Intercollegiate Climate Emergency Declaration and called for urgent action. We review waste production and the recyclability of surgical instrument packaging used in a common ear, nose and throat procedure (thyroidectomy) and suggest strategies to make this surgery more sustainable,. These strategies can be generalised to other surgeries.

**Methods:**

We prospectively audited packaging waste from 20 thyroidectomies performed at the Royal Marsden Hospital in the UK between July and December 2022. All packaging was weighed, categorised and analysed after the operation.

**Results:**

On average, each thyroidectomy produced packaging waste comprising 183g (34%) of plain paper/cardboard, 167g (31%) of soft plastic film, 142g (26%) of laminated paper, 37g (7%) of hard plastic and 11g (2%) of metal foil. Of all the packaging collected, only one item had a recycling label. When extrapolated to the 7,851 thyroidectomies performed in the National Health Service during the fiscal year 2021/2022, the estimated total weight of packaging waste would be 4.2 tonnes, of which only 31.4kg would be indicated as recyclable. When converted to an estimated carbon footprint, total carbon emissions would be 1,048kg CO_2_e, equivalent to three round trips from London to Edinburgh in a petrol car.

**Conclusion:**

This audit demonstrates the different categories and vast amount of packaging waste from a common operation. Manufacturers should place clear recyclability labels on packaging, and switch to recyclable materials and a digital information booklet where possible. Local waste audit and analysis can be simple first steps towards making surgery more sustainable.

## Introduction

The National Health Service (NHS), the largest publicly funded health system globally and the largest employer in Europe, has a crucial responsibility to lead the way in adopting emission-reduction measures and setting an example for others. The NHS accounts for approximately 4% of England’s total carbon footprint, emphasising the need for further action to minimise its impact on climate change.^1^

One significant area contributing to the NHS’s carbon footprint is the supply chain, which accounts for 59% of its total CO_2_ emissions. Within the healthcare system, operating theatres play a prominent role in generating carbon emissions owing to their resource-intensive nature.^2^ A systematic review revealed that a single operation can generate between 6 and 814kg of CO_2_e, equivalent to driving more than 2,000 miles in an average petrol car. The major contributors include the procurement of consumables, electricity usage and the use of anaesthetic gases.^3^

In November 2022, the three UK Surgical Royal Colleges issued an Intercollegiate Climate Emergency Declaration acknowledging that “climate change and anthropogenic environmental degradation pose a major threat to both human health and planetary health”.^4^ They called for urgent action and gave support to their members in bringing about changes within their own operating theatres.

Healthcare contributes more than 1% of all domestic waste in the UK, with operating theatres alone account for over 50% of all hospital waste, indicating the need for sustainable waste management practices.^5,6^

Disposable or single-use products used in surgical procedures have been identified as a significant factor contributing to the carbon footprint. In recent decades, the use of disposable equipment has been compounded by the increasing complexity of surgical systems and procedures. However, reliance on disposables lacks solid evidence of quality and safety benefits.^7^

Although altering the disposal practices and manufacturing materials of surgical instruments can be challenging, surgical instrument packaging that poses no biohazard risk and does not come into direct human contact offers an easy and potentially effective way in which to reduce surgical waste.

We reviewed the waste production and recyclability of surgical instrument packaging used in a common ear, nose and throat procedure (thyroidectomy). Between 2021 and 2022, 7,851 thyroidectomies were performed in the NHS alone, with more in the private sector. By reviewing this common standard procedure we hope to suggest steps that can be taken to reduce surgical waste, improve recycling and ultimately make surgery more sustainable.

## Methods

We prospectively audited surgical instrument and draping packaging waste from 20 thyroidectomies performed at the Royal Marsden Hospital, London, UK between July and December 2022. All thyroidectomies were performed by the senior surgeon (DK) with one assistant surgeon and a scrub nurse. The operating theatre was run by the same group of head and neck specialised scrub nurses and they were informed of the waste audit to facilitate the collection of packaging waste.

All operations were performed in similar fashion for the same indication with use of intraoperative nerve monitoring. Equipment is selected from a predefined operation-specific list and re-discussed at the theatre brief before each case (Table 1). For any variations or additional equipment used, the associated packaging was also included in the analysis. All packaging collected in the preparation room and throughout the operation was categorised, weighed and analysed after the operation. Recycling labels were recorded, if present. Packaging was categorised into “plain paper or cardboard”, “laminated paper”, “soft clear plastics”, “hard plastics” and “metal foil”. Each category was weighed in the operating theatre at room temperature (20°C) and 60% humidity. A digital weighing scale (Digi® DS-500, DIGI Europe Limited, UK) with a stated accuracy to 2g was used.

**Table 1:** Disposable packaging list of standard equipment from a thyroidectomy

Surgeons and scrub nurse gowns and gloves
Chlorhexidine for skin preparation
Diathermy set, tips and grounding pads
Nerve monitor electrodes
Drapes
Needles and syringes
Disposable skin flap retractors
Suction tubing and tip
Gauze and swabs
Sutures
Haemostatic metal clips
Light handles
Topical skin adhesive
Transparent film dressing
Standby items
Energy device (standby)
Haemostatic agent (standby)
Drain (standby)

Weights were averaged for each category and then extrapolated to estimate the annual weight of packaging waste for all thyroidectomies within the NHS in the UK using the national surgical registry, Hospital Episodes Statistics.^8^

## Results

**Figure 1:**
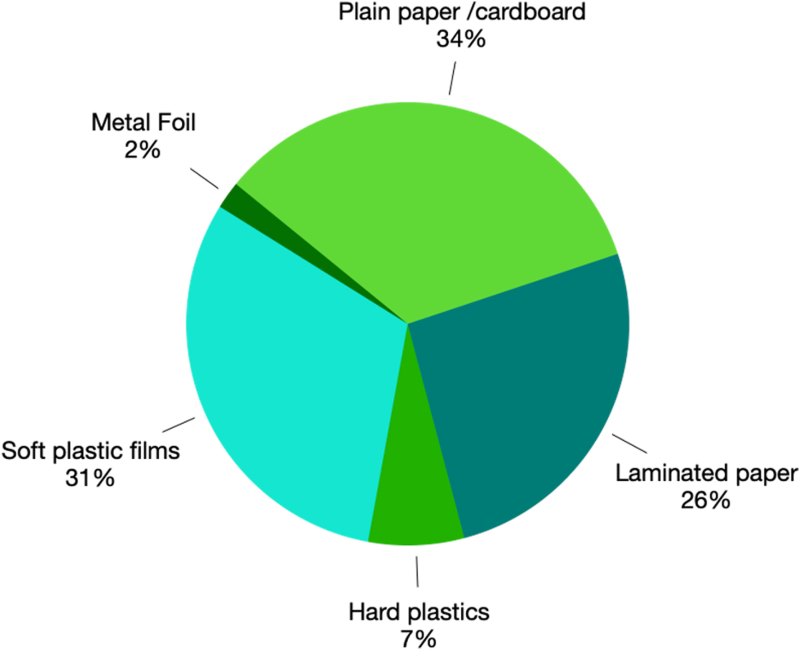
Percentage of categorised weight on average

The proportion of each categorised waste weight is shown in Figure 1.

The weight of surgical packaging from all 20 thyroidectomies was 10.8kg, of which 34% (3.7kg) was “plain paper/cardboard”, 31% (3.3kg) “soft plastic films”, 26% (2.8kg) “laminated paper”, 7% (0.7kg) “hard plastics” and 2% (0.2kg) “metal foil”. On average, each thyroidectomy produced packaging waste of 183g of plain paper/cardboard, 167g of soft plastic film, 142g of laminated paper, 37g of hard plastic and 11g of metal foil.

**Figure 2:**
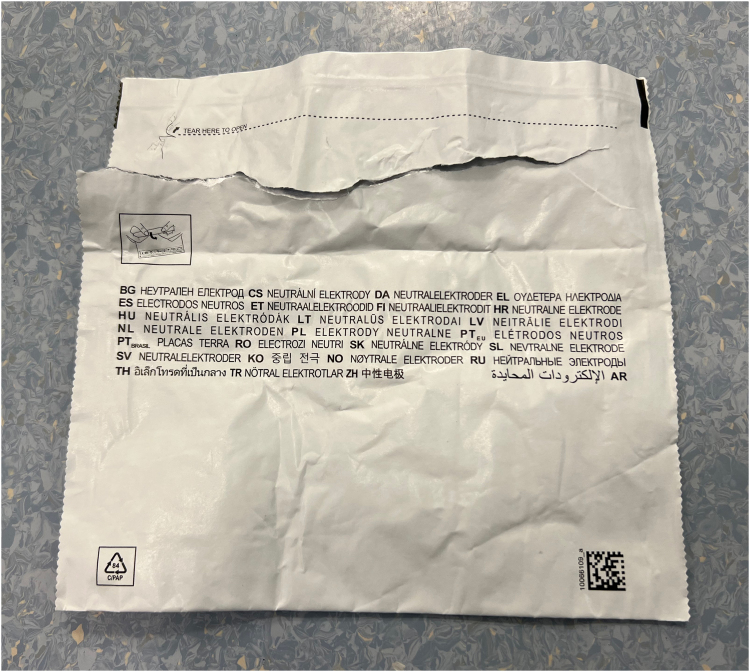
Packaging of the electrosurgical grounding plate

Of all the packaging items collected, only one (electrosurgical grounding plate) had a recycling label. It weighed 4g, accounting for only 0.7% of the total weight of all packaging. It was labelled with recycling code C/PAP 84, meaning “paper and cardboard/plastics/aluminium” (Figure 2).

In total, 7,851 thyroidectomies were performed during the fiscal year 2021/2022 in the NHS.^8^ Extrapolated to this, the estimated total packaging waste weight would be 4.2 tonnes, of which only 31.4kg would be indicated as recyclable. When converted to an estimated carbon footprint according to the methods of disposal, we found that footprint per tonne of hospital waste was lowest when it is recycled, releasing up to 65kg CO_2_e/tonne, whereas for low-temperature incineration of uncontaminated waste, carbon emissions were up to 249kg CO_2_e/tonne. This means the total carbon emissions extrapolated for the annual thyroidectomy-related packaging waste could be 1,048kg CO_2_e, which is equivalent to three round trips from London to Edinburgh in a petrol car.^9^

## Discussion

Thyroidectomy is one of the most common head and neck procedures performed worldwide. It is also widely understood and commonly practised, making it more relatable for surgeons across various specialties. In addition, thyroidectomy is a relatively straightforward operation that requires a standard set of equipment with few specialised instruments. This provides us with a more standardised dataset for analysis. Our results demonstrate that thyroidectomy produces a significant amount of waste from packaging alone. Changing the surgical instrument materials from the point of sustainability and recyclability may be challenging owing to issues related to instrument performance and human tissue contact, as well as the risk of biohazardous waste or contamination. Surgical instrument packaging, however, would be an easy place to start to reduce surgical waste without compromising patient care and has been a long-neglected area in research.

The NHS generated nearly 487,000 tonnes of direct waste in 2021/22, with only 16% of being recycled, 5% going directly to landfill and the rest being incinerated or subjected to alternative treatment.^10^ In 2021, the total percentage of all packaging waste recycled in the UK was 63%, whereas in our study less than 1% of our surgical instrument packaging waste was indicated as recyclable.^11^

Following discussion with the trust sustainability team and analysis by our local waste contractor, we were able to identify a number of areas for improvement.

1.  A high proportion of our “paper/cardboard” is easily recyclable in dry mixed recycling (DMR), although “laminated paper” is not recyclable.
2.  “Hard plastics” are also easily recyclable in DMR.
3.  For “soft plastic films”, we had a roughly 50/50 split of non-recyclable multilayer laminate films and recyclable mono-polyethylene laminate (mono-PE) films in our collected waste. Separating them and recycling only the mono-PE films would incur high costs for a specialised contractor with trained staff.
4.  Unlike household foil, our collected “metal foil” waste is multilayer laminated with plastic. It is for chemical recycling only, rather of DMR and requires separate collection.^12^

**Figure 3:**
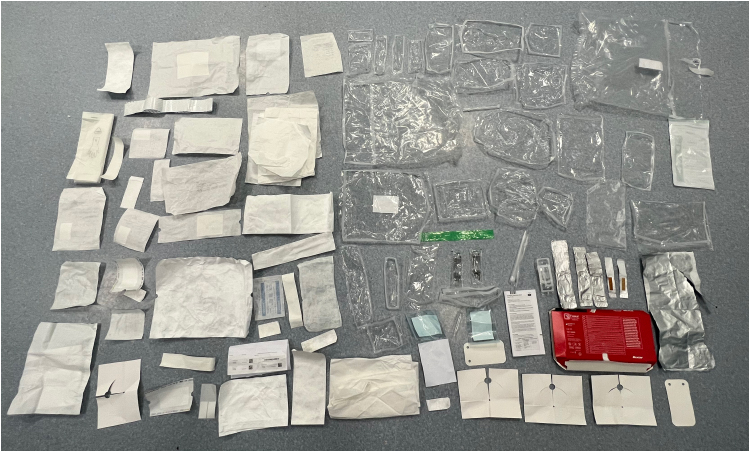
Example of packing waste used in single hemi-thyroidectomy operation

This study demonstrated the vast amount of packaging waste in surgery (Figure 3). Despite only one item of packaging having a recycling label, in its current form, 41% of the packaging we collected is easily recyclable, i.e. is plain paper/cardboard and hard plastic.

Potential improvements locally would involve working with the supply chain to reduce the use of non-recyclable material such as “laminated paper” and switch to “soft plastic films” made from recyclable mono-PE only. A separate collection of “metal foil” for specialised chemical recycling could be a solution with concerns about costs and genuine environmental benefits in chemical recycling.

Extrapolated to a larger scale, we make a number of proposals to improve the sustainability of surgical instruments.

1.  Manufacturers should declare the recyclability of packaging using clear universal labels. Use of clearer labels would reinforce recycling practices more easily with continuous education for our frontline healthcare members.
2.  Manufacturers should state what proportion of their packaging has been produced from recycled materials and have easily accessible information on the carbon footprint and sustainability of their devices.
3.  Manufacturers should reduce unnecessary packaging and additional information, such as booklets, should be digitised. One suggestion would be to bulk package instruments, especially when they are used in large quantities in a single operation; e.g. making sutures longer, packaging scrubs together in doubles or making operation-specific sets.
4.  Procurement teams in healthcare institutions should factor in the sustainability of packaging and products when purchasing equipment, work with manufacturers to reduce packaging waste by improving packaging efficiency and switch to recyclable materials where possible.
5.  Local waste analysis and discussions with sustainability management and the waste contractor should be undertaken to evaluate possible waste and promote recycling efficiency.
6.  Creation of standardised packaging, including at least common instruments such as suction tubing, drapes and gallipots.

Efforts to reduce the NHS’s carbon footprint must extend beyond individual hospitals and involve systemic changes throughout the healthcare sector. Collaboration between healthcare professionals, policy-makers and suppliers is crucial for implementing sustainable practices in the procurement of consumables and medical instruments. By leveraging its size and influence, the NHS can drive its emissions reductions, inspire other healthcare systems to follow suit and contribute to the global fight against climate change.

The proposals in this paper are an example of simple first steps to promote recycling and reduce usage. It is worth remembering that even perfect recycling is only one of the many steps in a broader sustainability framework. Other significant carbon contributors to the NHS include energy consumption, anaesthetic gases usage and travel within the NHS. Future research should explore a comprehensive approach to improve and address barriers to more sustainable practices to achieve a more holistic healthcare system. Surgeons, as key stakeholders in the healthcare system, have a unique opportunity to lead by example and drive the transition to a greener and more sustainable NHS. Through collective efforts, the healthcare sector can play a significant role in building a resilient and low-carbon future for the benefit of both current and future generations.

### Study limitations

As a single centre and surgeon study investigating a single operation, the results presented are specific to our practice. Nonetheless, our senior surgeon performs in excess of 150 thyroidectomies a year, and in a similar fashion to that frequently described in the literature. Furthermore, our results seek to demonstrate the recyclability of equipment that is commonly used for other procedures or all procedures, such as scrubs, drapes, needles and syringes. In addition, our study serves as an example for future packaging waste audits to be carried out in other centres and generalised to other surgeries, providing insights into how simple improvements can be sparked from a waste audit and can make surgeries more sustainable. We also only calculated the surgical packaging waste and did not include the anaesthetic contribution to waste production.

## Conclusions

To achieve a sustainable future, it is imperative that the healthcare industry plays an active role in advocating for and implementing sustainable practices. This includes promoting the judicious use of resources, exploring alternatives to disposable products, and advocating for the adoption of sustainable packaging solutions. Reducing the amount of packaging and improving recyclability is an easy and realistic first step in minimising surgical waste. This audit demonstrates the different categories and vast amount of packaging waste from a single, simple operation. A large proportion of the waste materials were easily recyclable but clear labels were lacking. Product manufacturers should place clear recyclability labels on packaging, switch to recyclable materials where possible and remove unnecessary information booklets that can be easily accessed digitally. Local waste audit and analysis can be simple steps to make surgery more sustainable and reduce the environmental impact while treating patients.
